# Quality assurance and validity of AI-generated single best answer questions

**DOI:** 10.1186/s12909-025-06881-w

**Published:** 2025-02-25

**Authors:** Ayla Ahmed, Ellen Kerr, Andrew O’Malley

**Affiliations:** University of St Andrews, St Andrews, UK

**Keywords:** Generative AI, Artificial Intelligence, ChatGPT, Assessment, LLM

## Abstract

**Background:**

Recent advancements in generative artificial intelligence (AI) have opened new avenues in educational methodologies, particularly in medical education. This study seeks to assess whether generative AI might be useful in addressing the depletion of assessment question banks, a challenge intensified during the Covid-era due to the prevalence of open-book examinations, and to augment the pool of formative assessment opportunities available to students. While many recent publications have sought to ascertain whether AI can achieve a passing standard in existing examinations, this study investigates the potential for AI to generate the exam itself.

**Summary of work:**

This research utilized a commercially available AI large language model (LLM), OpenAI GPT-4, to generate 220 single best answer (SBA) questions, adhering to Medical Schools Council Assessment Alliance guidelines the and a selection of Learning Outcomes (LOs) of the Scottish Graduate-Entry Medicine (ScotGEM) program. All questions were assessed by an expert panel for accuracy and quality. A total of 50 AI-generated and 50 human-authored questions were used to create two 50-item formative SBA examinations for Year 1 and Year 2 ScotGEM students. Each exam, delivered via the Speedwell eSystem, comprised 25 AI-generated and 25 human-authored questions presented in random order. Students completed the online, closed-book exams on personal devices under exam conditions that reflected summative examinations. The performance of both AI-generated and human-authored questions was evaluated, focusing on facility and discrimination index as key metrics.

**Summary of results:**

The screening process revealed that 69% of AI-generated SBAs were fit for inclusion in the examinations with little or no modifications required. Modifications, when necessary, were predominantly due to reasons such as the inclusion of "all of the above" options, usage of American English spellings, and non-alphabetized answer choices. 31% of questions were rejected for inclusion in the examinations, due to factual inaccuracies and non-alignment with students’ learning. When included in an examination, post hoc statistical analysis indicated no significant difference in performance between the AI- and human- authored questions in terms of facility and discrimination index.

**Discussion and conclusion:**

The outcomes of this study suggest that AI LLMs can generate SBA questions that are in line with best-practice guidelines and specific LOs. However, a robust quality assurance process is necessary to ensure that erroneous questions are identified and rejected. The insights gained from this research provide a foundation for further investigation into refining AI prompts, aiming for a more reliable generation of curriculum-aligned questions. LLMs show significant potential in supplementing traditional methods of question generation in medical education. This approach offers a viable solution to rapidly replenish and diversify assessment resources in medical curricula, marking a step forward in the intersection of AI and education.

## Introduction

The practice of active learning has been shown to increase examination performance among learners [1]. Falling under this category of study, retrieval practice has been proven to be an effective strategy to enhance meaningful learning [2]. More specifically, retrieval practice using single best answer (SBA) questions can greatly improve purposeful learning [3]. SBAs are renowned for their objectivity, efficiency, and ability to briefly encompass a wide range of knowledge [4]. They cultivate the development of critical thinking skills and the ability to make clinical decisions, not merely memorization [5]. Particularly with the use of case-based SBAs these questions facilitate the integration of theoretical knowledge with practical implementation, helping with the preparation for professional practice [6]. However, in the field of medical education, the quantity of SBAs available for formative use are scarce due to the challenges involved in producing them.

Best-practice guidelines have been established in the United Kingdom by the Medical Schools Council Assessment Alliance (MSCAA) to help examiners to produce SBAs that are of consistent high-quality [7]. A high-quality SBA should include a stem, lead-in and five options for candidates to choose from, comprising one correct answer and four distractors. Guidelines also advise on question tagging (to assist with examination blueprinting) and the use of abbreviations and reference ranges. For example, the stem—which contains the information needed to answer the question—must be in present tense and in third person narration. The lead-in question must avoid negative phrasing—such as “which is the least likely?” —and should be possible to answer without looking at the options. The five options should all be plausible answers, homogenous in length in relation to each other and presented in alphabetical order. Abbreviation guidelines include advise about chemical compounds and units of time/measurement. The guidelines also contain a detailed style guide relating to, for example, the use of apostrophes, notation of bacteria, capital letters, abbreviations etcetera [19]. These guidelines have been produced to foster consistency in the style and quality of SBAs across the United Kingdom ahead of the roll-out of national licencing examination, similar to what already exists in the United States in the Medical Licencing Examination (USMLE).

Producing an SBA of appropriate difficulty is also a challenge that examiners face. Questions should be challenging enough to discriminate between those who understand the material well and those who do not, however they should not be so difficult that they are discouraging or unaligned to the teaching provided [8]. To distinguish between higher and lower performing students, good SBAs should allow markedly better performance from those who tend score highly on exams than those who score poorly [9], in a concept known as ‘discrimination’. Moderately difficult items tend to demonstrate good discrimination, while very difficult and very easy SBA are more likely to show no discrimination or negative discrimination, whereby overall weaker students tend to do better than stronger students [10].

Creating SBAs requires medical knowledge, conceptual integration, and avoiding pitfalls [8]. Pitfalls can be identified by candidates with good examination technique (also known as “testwise” candidates) to occasionally correctly answer a question without possessing the underlying knowledge [11]. These pitfalls can include mutually exclusive distractors—where two mutually exclusive responses are correct—and the use of absolute terms such as: always, never, and all [12]. “Irrelevant difficulty” describes questions that are made difficult for reasons that are unrelated to the aim of the assessment [11]. This can arise from negatively phrased, long, and overly complicated questions [11]. Additionally, the process of constructing every SBA is a very time-consuming for medical educators [13]. Even with all of these factors being met, once a question is created—due to answer memorisation—reusing questions from year to year can threaten the validity, efficacy, and test security of exams [14]. This disposability further perpetuates the scarcity of SBAs. Although constructing ones owns SBAs can be effective, this exercise is unlikely to be met with enthusiasm due to it being unfamiliar and a perceived inefficient use of time [15].

Unique challenges were introduced during the Covid-19 pandemic in 2020 and affected several subsequent academic years. In order to ensure the safety of students and staff during examinations during the pandemic, most medical schools opted for online open-book examinations [16]. This decision resulted in vast numbers of SBAs essentially entering the public domain and reducing the number of questions available for use in subsequent years.

Recent advancements in generative AI offer potential solutions to the challenges associated with producing large numbers of SBA questions. Generative Pre-Trained Transformer (GPT) is a language model developed by OpenAI that powers ChatGPT, a chatbot app, which is designed to generate human-like text responses based on the information it receives from a user [17]. Due to their human-like text understanding and generation, OpenAI’s LLMs offer potential solutions to healthcare education [18, 19]. LLMs are trained to predict a sequence of fragments of words (i.e. ‘tokens’) based on the tokens, and their context, that come before them [20]. Therefore, LLMs can generate a novel sequence of words if trained on a sufficiently large amount of text data [20]. So far, this model has already been able to successfully pass the USMLE, so it is reasonable to hypothesize that the LLM could potentially write the exam itself [20]. While many recent publications have sought to ascertain whether AI can achieve a passing standard in existing examinations, this study investigates the potential for AI to generate the exam itself.

## Materials & methods

### Question generation

GPT-4 via ChatGPT, a commercially available AI LLM, was used to generate 220 SBA questions. A prompt (Table 1) was developed which incorporated abridged guidance from the MSCAA Style Guide, which was developed to define best practice in the style and format of SBA questions for the applied knowledge test (AKT) of the General Medical Council’s (GMC) upcoming Medical Licencing Examination (MLA). Also included in the prompt was an Intended Learning Outcome (ILO) from the case-based learning component of the Scottish graduate entry medical programme (ScotGEM) curriculum, which provided GPT-4 with the necessary context to generate the SBA. Examples of ILOs used in the prompt are included in Table 2. The prompt was presented (in January 2024) to independent instances of GPT-4 until 220 SBAs had been generated; this amounted to 74 iterations, which yielded 222 SBAs. The final two questions were omitted from the study leaving 220 SBA in total that were included in this study. Each SBA was recorded in preparation for quality assurance checks before potential inclusion in an examination.

**Table 1:** The prompt used to generate 220 SBA questions, based on abridged MSCAA guidance and learning outcomes that were addressed during the course

A good single best answer (SBA) question for medical students should have the following components: 1) A stem, which ensures the question is clinically relevant without the use of names for patients, bad practice/errors, setting of care (unless it influences decisions about correct answer), or any extraneous details, 2) A lead-in, which poses a specific question in which students can arrive at the correct answer without seeing the options and avoids negative phrasing or focus around bad practice, and 3) Five potential answers
There are some rules for the five potential answers: only one option should be correct; be relevant to the stem and lead-in; be plausible and realistic; the options must be listed in alphabetical order; neither "all of the above" or "none of the above" should be listed as options; be homogenous in content; there should always be five options
I will provide you with a learning outcome. You will write three good SBA questions for that learning outcome. You will also generate explanations for the correct answers to the questions
[The relevant ILO was inserted here]

**Table 2:** Examples of intended learning outcomes that were included in the prompt used to generate the questions

Describe the micro- and macroscopic structure and function of muscles, bones and joints
Compare and contrast Type 1 and Type 2 hypersensity reactions
Explain how O2 and CO2 are transported in the blood, and relate this to the structure of haemoglobin
Discuss how the concept of 'Realistic Medicine' may be applied in practice, with particular reference to the care of the older adult
Discuss the clinical features, investigation and management of chronic obstructive airways disease

### Quality assurance

The AI-generated SBAs underwent standard quality-assurance screening to ensure compliance with the stipulated guidelines and ILOs according to our standard assessment process. Each SBA was sent to the faculty member responsible for the ILO that was used in its generation, who assessed each question for suitability, alignment and quality. ​ Questions were categorised as either acceptable, modifiable, or rejected. The reason for modification/rejection was recorded and categorised. Finally, all questions included in the examination were assessed by a broader panel of 8 faculty members (clinical and non-clinical) to ensure consistency and balance.​ All staff involved in this quality assurance process were familiar with the MSCAA guidance and had received training on SBA writing.

### Examination

A subset of questions that were identified as either acceptable or modifiable were selected (*n* = 50) and used alongside an equal number of human-authored questions (*n* = 50) to construct two formative SBA examinations each of 50 items (one for Year 1 and one for Year 2 of the ScotGEM programme, with 25 AI-authored questions and 25 human-authored questions each), which were subsequently undertaken by medical students. A check of the paper was conducted to ensure no errors or amendments had inadvertently been introduced following the quality-assurance process.

Both examinations were delivered online via the Speedwell eSystem platform within a set time, following the usual process for the delivery of formative examinations. Students used their own devices to complete the formative examinations and could do so in a location of their choosing within the given time. Students were encouraged to complete the formative examination as a closed book exercise to better prepare for their in-person summative closed book examinations and give a better indication of their learning. The order of the questions, both AI generated and human-authored, were randomised in both examinations so that neither were grouped together. After the opportunity to undertake the formative examinations had closed, results and marking keys were released to students on the next working day. A feedback session on the overall performance in the formative examinations was also provided to both year groups in the week that results were released.

### Post-hoc item analysis

For each question facility (F) was calculated. Facility indicates the proportion of student responses that were correct and is therefore occasionally referred to as "difficulty". A value of 0 means that no students answered the question correctly, while a value of 1 means that all students answered the question correctly. This was done by taking the sum of the actual marks (1 or 0) for each student and dividing this by the number of candidates [21].

For each question discrimination index (DI) was also calculated. This was done by subtracting the facility score calculated from the worst-performing 27% of students from the facility score of the best-performing 27% of students. These groups are categorized based on students' overall examination performance. DI therefore enables assessors to discern whether a given question is effective at separating out the best- and worst- performing students.

A positive DI means that more of the best-performing students chose the correct answer than in the worst-performing students. A DI of 0 means the best-performing and worst-performing students did equally well (or badly) at that question. A negative DI means that more of the worst-performing students selected the correct answer than those in the best-performing group. Items with a negative DI could indicate a problem with the question, such as a technical error (e.g. an incorrect answer is labelled as the correct answer in the assessment software) or an issue with alignment between the items and the students' learning [21]. For the purposes of this study, an acceptable DI was considered to be > 0.2 since this was a formative examination, however a DI of > 0.3 would normally be required in a summative examination.

The performance of both AI-generated and human-authored questions was evaluated by comparing the F and DI scores of human- and AI-authored SBAs, and t-tests for each measurement (F and DI) was conducted between the AI-generated vs human authored questions to ascertain if any significant difference existed between the questions.

### Ethical considerations

Ethical approval was awarded on 19 Oct 2024 by the School of Medicine Ethics Committee at University of St Andrews (Reference number MD17293). Since this study does not involve patients a clinical trial number was not applicable. All students received information about the study before attempting the exam, and a consent form was required to be completed. Students’ exam responses were only used in this study if they provided consent. These provisions aligned our study with the Declaration of Helsinki. As there was a dependent relationship between the researcher (i.e. teachers/assessors on the ScotGEM programme) and the students, it was made clear that withholding consent would not disadvantage the student and that they would be able to attempt the exams as normal.

Students’ individual responses were not anonymised to provide them with feedback after the conclusion of the exam; however, the identities of the students were not presented to the research team for the purposes of the post-hoc analysis. Participants were provided with their induvial exam feedback (privately) and the general findings of this study (during a whole-class briefing session). Students’ responses and were stored securely on University cloud storage (OneDrive) and only accessible by the research team. Anonymised datasets used and/or analysed during the current study are available from the corresponding author on reasonable request.

## Results

The total number of participants (i.e. students who undertook the exam and consented to their data being included in this study) was 142, comprising 84 from Year 1 and 58 from Year 2.

### Quality assurance

**Fig. 1:**
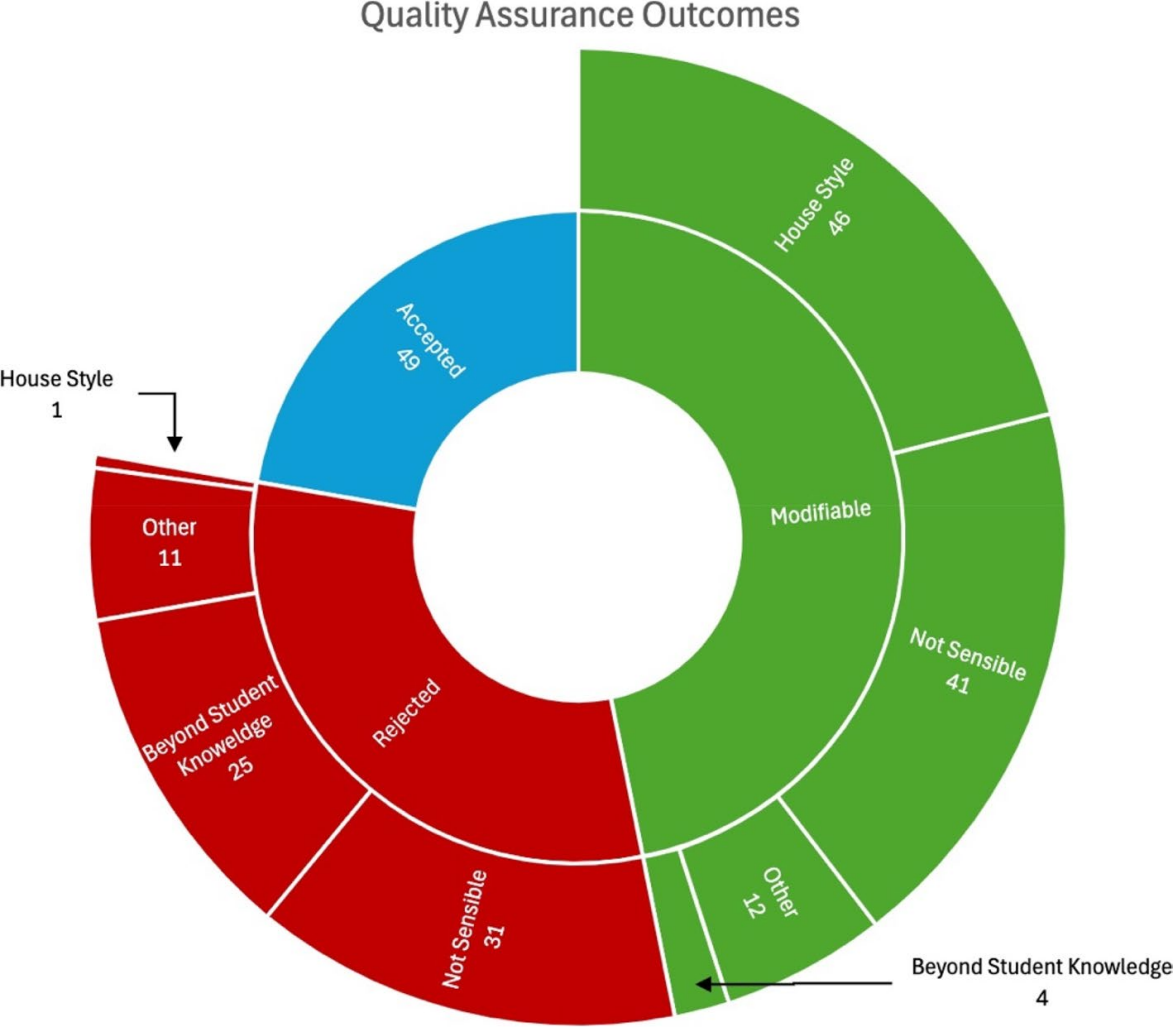
Outcomes of the quality assurance assessment of the 220 AI-generated SBA questions

Of the 220 SBA questions generated by GPT-4, 49 (22.2%) were usable without any amendments whatsoever, 103 (46.8%) required minor modifications to correct issues of style, content or alignment, and 68 (30.9%) were rejected because they were either unsalvageable or would have required prohibitively extensive amendments to enable their inclusion in an examination. The reasons for rejection or modification of a question were categorized into: “beyond student knowledge”, “improper house style”, “not sensible”, and “other”, which included items that were too simple, duplicates, or not items that were not aligned to the provided learning outcome. These findings are included in Fig. 1.

### Beyond student knowledge

These questions did not align with student learning. This included information that was either not taught in a lecture, taught in teaching that took place after the exam, or not in the medical school curriculum at all. A rejected example includes the question below that mentions the respiratory system, which had not been taught at this point:

- A 25-year-old man presents to his GP with a fever and a productive cough. A chest x-ray reveals consolidation in the right lower lobe.
- Which of the following is the most likely immune response to this infection?
  A Activation of B cells to produce antibodies
  B Activation of cytotoxic T cells
  C Activation of natural killer cells
  D Phagocytosis by neutrophils
  E Release of interferons by infected cells

### House style

These questions involved failure to abide to the format required for medical school questions as outlined by the MSCAA Style Guide [7]. Although these guidelines were incorporated into the prompt inputted into the AI, occasionally mistakes were still made by the model. These mistakes included: incorrect wording that does not affect the answer, unnecessary addition of information, an option being “all/none of the above”, a “NOT” question and Americanised spelling. Often, these questions abided to the other guidelines and were, therefore, easily modifiable and eligible for acceptance. A modifiable example includes a “NOT” in the question:

- A 65-year-old patient is admitted to the hospital with an acute confusional state.
- Which of the following is NOT a recommended management option for this patient?
  A Administering antipsychotic medication
  B Ensuring adequate hydration and nutrition
  C Providing reality orientation
  D Using bed alarms
  E Using physical restraints

### Not sensible

This category encompasses all the questions that are inherently confusing for the student to answer. This includes questions that do not make sense, are factually incorrect, have multiple correct/similar options, do not pass the “cover test” which defines the ability to arrive at the correct answer without looking at the options, have incorrect answers, contain incorrect terminology that does affect the answer, are extremely vague, or have missing/incorrect crucial information (e.g. reference ranges). Sometimes questions were modified to ensure guideline adherence, but some were also rejected entirely. A modifiable example includes the lack of reference ranges for PaCO2 and pH, both required to correctly answer the question:

- A 60-year-old woman with a history of chronic obstructive pulmonary disease (COPD) presents to her primary care physician with worsening shortness of breath. Her arterial blood gas shows a PaCO2 of 60 mmHg and a pH of 7.30.
- What is the primary mechanism by which CO2 is transported in the blood?
  A As bicarbonate ions
  B As carbamino compounds
  C Bound to albumin
  D Bound to haemoglobin
  E Dissolved in plasma

### Other

Too Simple: A few questions generated—although correct and fully adhered to the guidelines—were too simple for the medical school level. This also entails questions in which the correct answer was mentioned somewhere in the question. A rejected example includes a question that is too easy:

- A 45-year-old female presents to her GP with fatigue, pallor, and shortness of breath. Her blood tests show a low haemoglobin level.
- What is the most likely diagnosis?
  A Anaemia
  B Asthma
  C Chronic obstructive pulmonary disease
  D Pneumonia
  E Tuberculosis

Repeat: This category entails questions that are so similar they are essentially repeats. One learning outcome is meant to generate three different questions. In these questions, one learning outcome led to the generation of two or more very similar—borderline exact—questions.

Does not align to the ILO: A few questions did not test the LO given. A rejected example is this question that asked for specific first-line treatment for Type 2 diabetes although the learning outcome was “Be aware of UK medicine legislation and principles of safe, effective, and sustainable prescribing”. This question was also incorrect as there is no UK Medicines Legislation that determines first line choice of treatments:

- A 45-year-old man with a history of hypertension presents to his GP with a new diagnosis of type 2 diabetes mellitus.
- According to UK medicine legislation, which of the following is the most appropriate first-line treatment for this patient?
  A Gliclazide
  B Glimepiride
  C Metformin
  D Pioglitazone
  E Sitagliptin

### Post-hoc Item analysis

#### Facility

**Fig. 2:**
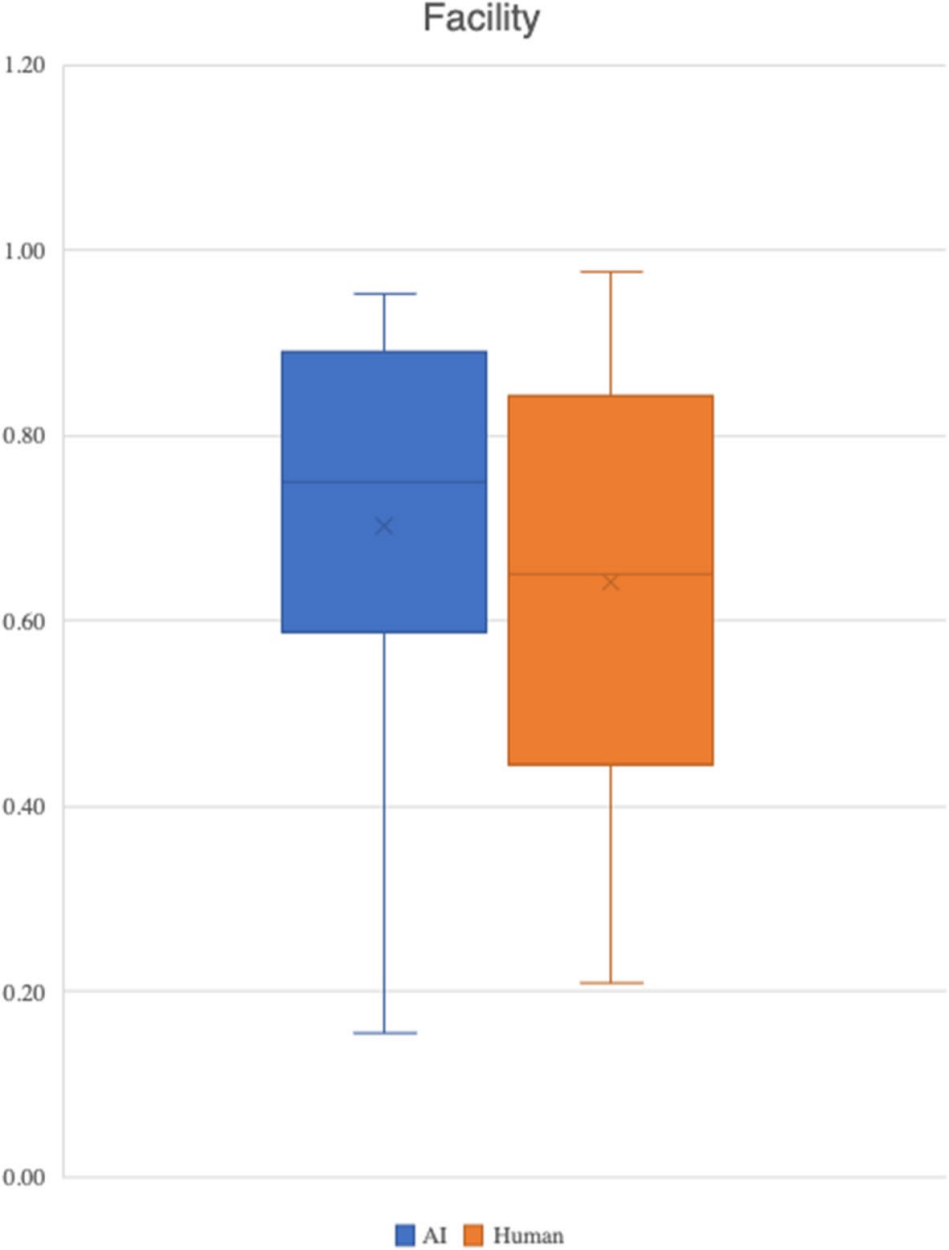
Facility calculations for AI-generated and human-authored questions

There was no statistically significant difference in facility between AI-generated and human authored questions (*p* = 0.176)​. However, descriptive statistics suggest that students found the AI-generated questions easier than human authored ones.​ These results are summarised in Fig. 2 and Table 3.

**Table 3:** Descriptive statistics of facility for human-authored and AI-generated questions

	**AI-generated SBAs**	**Human-authored SBAs**
**Mean**	0.70	0.64
**Minimum**	0.15	0.21
**Maximum**	0.95	0.97

#### Discrimination index

**Fig. 3:**
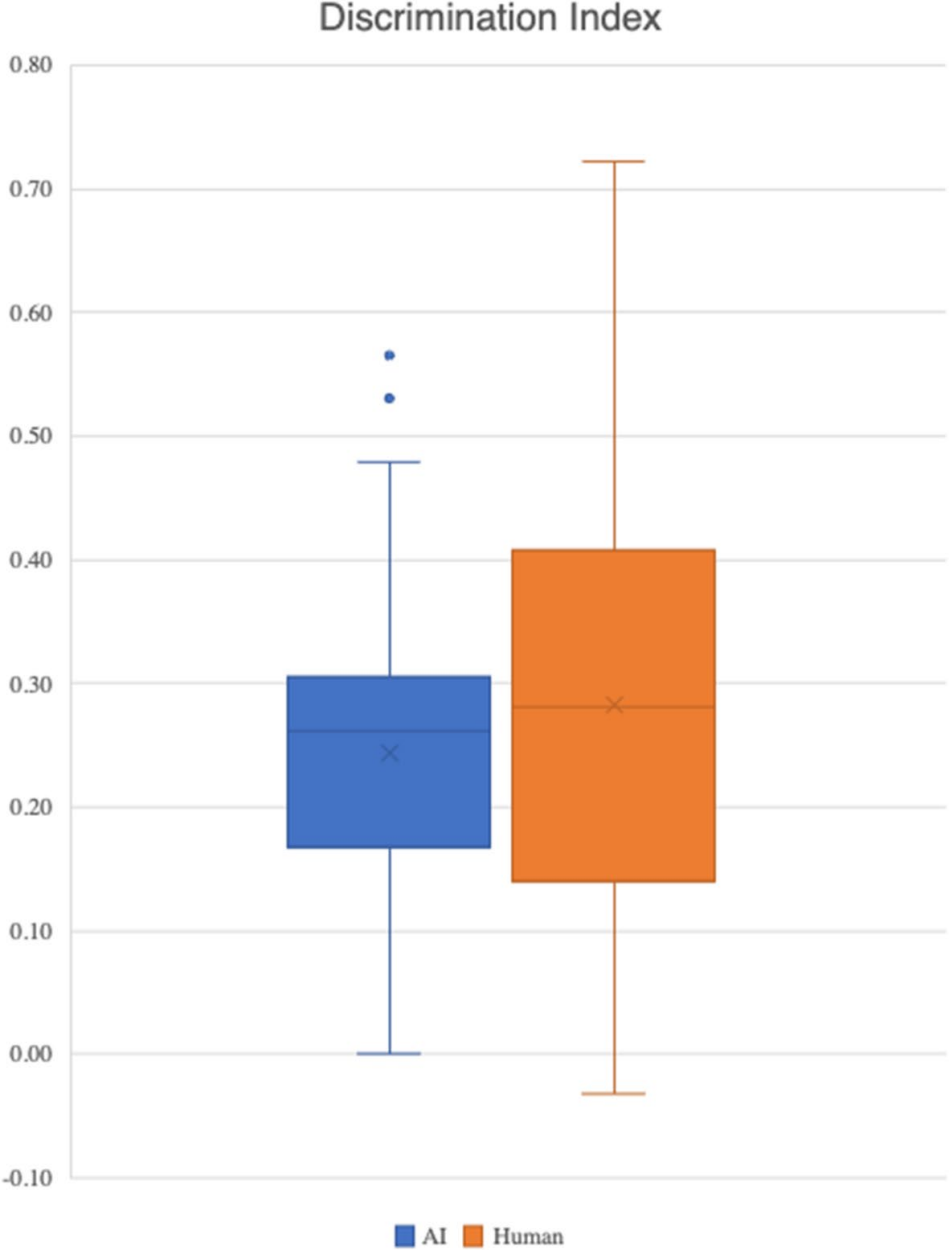
Discrimination index calculations for AI-generated and human-authored questions

There was no statistically significant difference in discrimination index between AI-generated and human-authored questions (*p* = 0.175)​. However, because facility was slightly higher in AI-authored questions (0.70 vs 0.64), they were less discriminating.​ These results are summarised in Fig. 3 and Table 4.

**Table 4:** Descriptive statistics of discrimination index for human-authored and AI-generated questions

	**AI-generated SBAs**	**Human-authored SBAs**
**Mean**	0.24	0.28
**Minimum**	0.00	−0.03
**Maximum**	0.56	0.72

## Discussion

The outcomes of this study suggest that AI LLMs can generate SBA questions that are in line with best-practice guidelines and specific ILOs, showing significant potential in supplementing traditional methods of question production in medical education. While 69% of questions were usable with no or minor modification, 31% of questions were not suitable for inclusion; these findings highlight the necessity of a systematic quality assurance process to ensure only high-quality items proceed into students’ examinations. Issues primarily relate to formatting/style, absent constructive alignment and inappropriate level of difficulty.

When quality-assured AI-generated questions are used in examinations, descriptive statistics suggest that AI-generated questions are slightly easier and less discriminating that human-authored questions, although not to a statistically significant degree. Of the 50 human-authored questions, 33 had a DI > 0.2 and 24 had a DI > 0.3; among the 50 AI-generated questions, 32 achieved a DI > 0.2 and 12 achieved a DI > 0.3. These findings suggest that, as higher levels of item discrimination are sought, the benefits of using AI to generate questions may become more limited.

Although there is a paucity of literature in this emerging area, the findings of this study broadly align with early reports elsewhere in the literature. There is broad agreement that models can generate questions that are often indistinguishable from human-written ones [22–26], there is also trepidation regarding the quality of the AI-generated questions. Although this study did not detect a statistical difference in discrimination index between AI- and human- generated questions, other reports in the literature suggest this difference does exist in that AI-generated questions may have lower discriminatory power compared to human-written questions [24, 27].

In addition to concerns around quality, there are also emerging reports in the literature regarding outdated terminology, age- and gender- specific inaccuracies, and geographically insensitivities being detected in AI-generated examination questions [23]. Similar issues relating to representation have also been detected when creating other types of content involving patients or clinical scenarios [28–30], and also when using generative AI to assist practitioners with clinical reasoning [31].

These findings suggest that complete replacement of human-authored questions is not feasible. However, there is considerable potential for the use of this technology to assist humans. This approach offers a viable solution to rapidly replenish and diversify assessment resources in medical curricula, marking a step forward in the intersection of AI and education. Even when AI-generated questions do not satisfy the high-standards demanded by Universities and regulators, they can still serve to inspire new ideas for human authors. A portion of the process in producing questions involves the creative aspect of curating a stem, question, and 5 options. Even if a question is entirely rejected and re-written—not only modified—the initial ideas can be of great help. In this way, the AI can essentially aid in solving writer’s block.

Due to the infancy and fast-moving capabilities of generative AI tools, there are some limitations associated with this study that could be overcome as the technology develops. Possible approaches to refining our method includes using more specific ILOs when inputting our prompt into the LLM. A common complaint of students is the vagueness of the ILOs, which can complicate determining which facts are important to focus on. This distinguishment could be beneficial in a course such as medicine, where the volume of content is extremely large. An alternative—or addition—to this could be to provide the LLM with actual teaching materials or lecture recordings. This could generate questions that are better aligned with students’ learning. Due to the pre-determined assessment format and finite number of participants a pre-requisite sample size was not calculated a priori; this represents a limitation of this study, which likely would benefit from more assessment items and participants.

In terms of the LLM used, the exponential advancement of AI could potentially generate a more sophisticated model that could be incorporated instead as previously mentioned. Additionally, we could append our own question banks to train our own model. The simplicity of a specialized model could be used for scaling up the use of this technology. This technology could also display adaptive difficulty where questions can be adjusted in difficulty based on the student’s performance, ensuring appropriate levels of challenge. With sufficient trial and error, a fully trained model could be released to the public for student and teacher use.

While this study focused on the development of SBAs, other forms of assessment used in medical teaching can be evaluated. This includes Short Written Answers (SWAs), Very Short Answer Questions (VSAQs), and Objective Structural Clinical Examinations (OSCEs). While these, and the SBAs, can be used in the production of formative questions there is also a possibility that this technology could be used in summative assessment as well.

Focus groups of both students and staff could potentially highlight the direction this research could go in. By discovering the perspectives of student and staff on what they thought of the study, this could reveal information about where the data should be applied.

## Conclusion

The outcomes of this study suggest that AI LLMs can generate SBA questions that are in line with best-practice guidelines and specific LOs. However, the necessity of a quality assurance process to fine-tune formatting and curriculum alignment is evident. When quality-assured AI-authored questions are used in exams, they do not perform any differently to human-authored questions. The insights gained from this research provide a foundation for further investigation into refining AI prompts, aiming for a more reliable generation of curriculum-aligned questions.

## Data Availability

Anonymised datasets used and/or analysed during the current study are available from the corresponding author on reasonable request.

## Abbreviations

AKT: Applied knowledge test
AI: Artificial intelligence
DI: Discrimination index
F: Facility
GMC: General medical coucil
GPT: Generative pretrained transformer
ILO: Intended learning outcome
LLM: Large language model
MSCAA: Medical schools council assessment alliance
OSCE: Objective structure clinical examination
SBA: Single best answer
ScotGEM: Scottish graduate entry medicine
SWA: Short written answer
USMLE: United States medical licencing examination
vSAQ: Very short written answer question

## References

[1] Freeman S, Eddy SL, McDonough M, Smith MK, Okoroafor N, Jordt H, et al. Active learning increases student performance in science, engineering, and mathematics. Proc Natl Acad Sci U S A. 2014;111(23):8410–5. 24821756 10.1073/pnas.1319030111 PMC4060654

[2] Karpicke JD, Blunt JR. Retrieval practice produces more learning than elaborative studying with concept mapping. Science. 2011;331(6018):772–5. 21252317 10.1126/science.1199327

[3] Smith MA, Karpicke JD. Retrieval practice with short-answer, multiple-choice, and hybrid tests. Memory. 2014;22(7):784–802. 24059563 10.1080/09658211.2013.831454

[4] Mujeeb AM, Pardeshi ML, Ghongane BB. Comparative assessment of multiple choice questions versus short essay questions in pharmacology examinations. Indian J Med Sci. 2010;64(3):118–24. 22569324

[5] Bassett MH. Teaching Critical Thinking without (Much) Writing: Multiple-Choice and Metacognition. Teach Theol Relig. 2016;19(1):20–40.

[6] Khan MU, Aljarallah BM. Evaluation of Modified Essay Questions (MEQ) and Multiple Choice Questions (MCQ) as a tool for Assessing the Cognitive Skills of Undergraduate Medical Students. Int J Health Sci (Qassim). 2011;5(1):39–43. PMC3312767 22489228

[7] Medical Schools Council Assessment Alliance. Applied Knowledge Test Style Guide (v2). 2022.

[8] Artsi Y, Sorin V, Konen E, Glicksberg BS, Nadkarni G, Klang E. Large language models for generating medical examinations: systematic review. BMC Med Educ. 2024;24(1):354. 38553693 10.1186/s12909-024-05239-y PMC10981304

[9] Kumar D, Jaipurkar R, Shekhar A, Sikri G, Srinivas V. Item analysis of multiple choice questions: A quality assurance test for an assessment tool. Med J Armed Forces India. 2021;77(Suppl 1):S85–9. 33612937 10.1016/j.mjafi.2020.11.007 PMC7873707

[10] Sim SM, Rasiah RI. Relationship between item difficulty and discrimination indices in true/false-type multiple choice questions of a para-clinical multidisciplinary paper. Ann Acad Med Singap. 2006;35(2):67–71. 16565756

[11] Coughlin PA, Featherstone CR. How to write a high quality Multiple Choice Question (MCQ): a guide for clinicians. Eur J Vasc Endovasc Surg. 2017;54(5):654–8. 28870436 10.1016/j.ejvs.2017.07.012

[12] Rush BR, Rankin DC, White BJ. The impact of item-writing flaws and item complexity on examination item difficulty and discrimination value. BMC Med Educ. 2016;16(1):250. 27681933 10.1186/s12909-016-0773-3 PMC5041405

[13] Gilardi F, Alizadeh M, Kubli M. ChatGPT outperforms crowd workers for text-annotation tasks. Proc Natl Acad Sci U S A. 2023;120(30):e2305016120. 37463210 10.1073/pnas.2305016120 PMC10372638

[14] Leo J, Kurdi G, Matentzoglu N, Parsia B, Sattler U, Forge S, et al. Ontology-based generation of medical, multi-term MCQs. Int J Artif Intell Educ. 2019;29(2):145–88.

[15] Palmer E, Devitt P. Constructing multiple choice questions as a method for learning. Ann Acad Med Singap. 2006;35(9):604–8. 17051275

[16] Monaghan AM. Medical Teaching and Assessment in the Era of COVID-19. J Med Educ Curric Dev. 2020;7:238212052096525. 10.1177/2382120520965255 PMC7573748 33117891

[17] Plevris V, Papazafeiropoulos G, Jiménez Rios A. Chatbots Put to the Test in Math and Logic Problems: A Comparison and Assessment of ChatGPT-3.5, ChatGPT-4, and Google Bard. Ai. 2023;4(4):949–69.

[18] Gordon M, Daniel M, Ajiboye A, Uraiby H, Xu NY, Bartlett R, et al. A scoping review of artificial intelligence in medical education: BEME Guide No. 84. Med Teach. 2024;46(4):446–70. 10.1080/0142159X.2024.2314198 38423127

[19] Aster A, Laupichler MC, Rockwell-Kollmann T, Masala G, Bala E, Raupach T. ChatGPT and other large language models in medical education — scoping literature review. Medical Science Educator. 2024. 10.1007/s40670-024-02206-6. 10.1007/s40670-024-02206-6 PMC11933646 40144083

[20] Kung TH, Cheatham M, Medenilla A, Sillos C, De Leon L, Elepano C, et al. Performance of ChatGPT on USMLE: Potential for AI-assisted medical education using large language models. PLOS Digit Health. 2023;2(2): e0000198. 36812645 10.1371/journal.pdig.0000198 PMC9931230

[21] Kelley TL. The selection of upper and lower groups for the validation of test items. J Educ Psychol. 1939;30(1):17–24.

[22] Bedi S, Fleming SL, Chiang C-C, Morse K, Kumar A, Patel B, et al. QUEST-AI: a system for question generation, verification, and refinement using AI for USMLE-Style Exams. medRxiv. 2024. 10.1101/2023.04.25.23288588. 10.1142/9789819807024_0005 39670361

[23] Klang E, Portugez S, Gross R, Kassif LR, Brenner A, Gilboa M, et al. Advantages and pitfalls in utilizing artificial intelligence for crafting medical examinations: a medical education pilot study with GPT-4. BMC Med Educ. 2023;23(1):772. 37848913 10.1186/s12909-023-04752-w PMC10580534

[24] Laupichler MC, Rother JF, Grunwald Kadow IC, Ahmadi S, Raupach T. Large language models in medical education: comparing ChatGPT- to human-generated exam questions. Acad Med. 2024;99(5):508–12. 38166323 10.1097/ACM.0000000000005626

[25] Zuckerman M, Flood R, Tan RJB, Kelp N, Ecker DJ, Menke J, et al. ChatGPT for assessment writing. Med Teach. 2023;45(11):1224–7. 37789636 10.1080/0142159X.2023.2249239

[26] Kiyak YS, Emekli E. ChatGPT prompts for generating multiple-choice questions in medical education and evidence on their validity: a literature review. Postgrad Med J. 2024;100(1189):858–65. 38840505 10.1093/postmj/qgae065

[27] Coşkun Ö, Kıyak YS, Budakoğlu Iİ. ChatGPT to generate clinical vignettes for teaching and multiple-choice questions for assessment: a randomized controlled experiment. Med Teach. 2025;47(2):268–74. 10.1080/0142159X.2024.2327477. 10.1080/0142159X.2024.2327477 38478902

[28] O’Malley A, Veenhuizen M, Ahmed A. Ensuring appropriate representation in artificial intelligence-generated medical imagery: protocol for a methodological approach to address skin tone bias. JMIR AI. 2024;3:e58275. 39602221 10.2196/58275 PMC11635324

[29] Fan BE, Chow M, Winkler S. Artificial Intelligence-Generated Facial Images for Medical Education. Med Sci Educ. 2023;34(1):5–7. 2023. 10.1007/s40670-023-01942-5. 10.1007/s40670-023-01942-5 PMC10948638 38510393

[30] Ali R, Tang OY, Connolly ID, Abdulrazeq HF, Mirza FN, Lim RK, et al. Demographic representation in 3 leading artificial intelligence text-to-image generators. JAMA Surg. 2024;159(1):87–95. 37966807 10.1001/jamasurg.2023.5695 PMC10782243

[31] M'gadzah SAT, O'Malley A Does a complex prompt alter the diagnostic accuracy of common ophthalmological conditions by GPT-4? JMIR Preprints. 10.2196/preprints.64986.

